# The effect of live-performed music therapy with physical contact in preterm infants on parental perceived stress and salivary cortisol levels

**DOI:** 10.3389/fpsyg.2024.1441824

**Published:** 2024-10-07

**Authors:** Monia Vanessa Dewan, Miriam Ader, Tim Kleinbeck, Anne-Kathrin Dathe, Manfred Schedlowski, Harald Engler, Ursula Felderhoff-Mueser, Nora Bruns, Susann Kobus

**Affiliations:** ^1^Department of Paediatrics I, University Hospital, University of Duisburg-Essen, Essen, Germany; ^2^Center for Translational Neuro- and Behavioural Sciences, C-TNBS, Faculty of Medicine, University Duisburg-Essen, Essen, Germany; ^3^Department of Health and Nursing, Occupational Therapy, Ernst-Abbe-University of Applied Sciences Jena, Jena, Germany; ^4^Institute of Medical Psychology and Behavioral Immunobiology, University Hospital Essen, University of Duisburg-Essen, Essen, Germany; ^5^Center of Artistic Therapy, University Medicine Essen, Essen, Germany

**Keywords:** music therapy, preterm infant, cortisol, stress reduction, NICU, parents, parental stress

## Abstract

**Introduction:**

Parents of preterm infants face a stressful life event which might have long term impact on the parent–child relation as well as on the infant’s cognitive and socio-emotional development. Both music therapy (MT) and physical contact (PC) are stress-reducing interventions for parents and preterm infants on the neonatal intensive care unit (NICU). Meanwhile, especially close PC is considered as standard care (SC) in most NICUs. However, the effect of live performed MT with PC on parental perceived stress and cortisol levels has barely been investigated. We hypothesized that MT with PC leads to reduced stress levels and lower salivary cortisol concentrations compared to SC in parents of preterm infants during the first 4 weeks after birth.

**Methods:**

Randomized-controlled trial enrolling the parents of 99 preterm infants (MT *n* = 50, SC *n* = 49 infants). The infants received either MT with PC or SC only. Perceived stress was measured with the perceived stress questionnaire 20 (PSQ-20) after birth and 4 weeks later. Salivary cortisol levels were obtained and measured weekly after birth for 4 weeks.

**Results:**

Forty-two mothers and eight fathers of the intervention group (MT with PC) as well as *n* = 43 mothers and *n* = 6 fathers of the control group (SC) were enrolled. For the intervention group, salivary cortisol was reduced 4 weeks after birth [mothers 5.5 nmol/l (confidence interval (CI) 3.6–7.5); fathers 8.3 (CI 7.2–9.4)] compared to the control group [mothers 10.3 nmol/l (CI 5.4–15.3); fathers 14.8 (CI 8.9–20.7)]. Overall perceived stress scores decreased in the intervention group (mothers −17.6; fathers −12.6) and increased in the control group (mothers +6.1; fathers +21.4) over 4 weeks.

**Discussion:**

Live-performed MT with PC in preterm infants might be an effective, non-invasive intervention to reduce parental stress and cortisol levels. Future studies should investigate the long-term effects of this intervention on the parent-infant relation as well as on the infants’ cognitive and socio-emotional development.

**Clinical trial registration:**

https://drks.de/search/en/trial/DRKS00025755 identifier [DRKS00025755].

## 1 Introduction

Prematurity is defined as birth before 37 weeks’ gestation. Worldwide more than 1 in 10 infants are born premature (Ohuma et al., 2023). As preterm birth coincides with a vulnerable period of brain development, preterm infants are at increased risk of neurodevelopmental impairments in later life. The risk increases with decreasing gestational age (Marlow et al., 2014; Wallois et al., 2020). Stress caused by, e.g., painful procedures, noise and parental separation has been identified as harmful factors for brain development (Cook et al., 2023; Cong et al., 2017; Zhang et al., 2022). A stress reducing, non-invasive intervention is music therapy (MT), which has gained attention in recent years (Yue et al., 2021). Previous studies showed that live and individually performed MT has positive effects on the preterm infants’ vital parameters, feeding behavior and weight gain (Yue et al., 2021; Erdei et al., 2024; Kobus et al., 2021; Kraft et al., 2021). While the long-term effects of MT on clinical neurodevelopment are still under investigation, results from diffusion tensor imaging and resting-state functional imaging studies at term equivalent age hint at potential effects of MT on neurodevelopment (Sa de Almeida et al., 2020; Haslbeck et al., 2020, 2021; Dewan et al., 2024).

Not only the preterm infant faces stressful events during the treatment on the neonatal intensive care unit (NICU), but also their parents undergo a critical life event (Weigl et al., 2020; Lundqvist et al., 2019). Parents of preterm infants are also at increased risk of parental separation (Nusinovici et al., 2017). Preterm birth interrupts bonding, which negatively impacts the development of the mother–child-relationship and might have substantial effects on the quality of early infant-parent-interaction (Fernández Medina et al., 2018). Postnatal bonding is important for the infant’s social, emotional and cognitive development and has positive impact on later mental health and resilience (Winston and Chicot, 2016). Following a study by Rusanen et al., postnatal bonding problems are related to socio-emotional problems in children at 2 years (Rusanen et al., 2024).

As stress negatively impacts bonding (Khoramirad et al., 2021) promoting parental well-being during the NICU stay is an important issue in developmental care for preterm infants. A promising family-centerd intervention is music therapy (MT), which is live performed by a certified music therapist (Haslbeck et al., 2023). Recent studies showed that MT reduces maternal distress, anxiety and depressive symptoms (Kehl et al., 2020; Kobus et al., 2022a; Kraft et al., 2021). It also positively modulates the mothers’ perception of the infant (Kobus et al., 2023).

Besides MT, the beneficial effects of physical contact (PC) between preterm infants and their parents/caregivers have been recognized and many NICUs all over the world have adopted the save practice of PC contact in developmental care for preterm infants (Bedetti et al., 2023). While most studies refer to close PC, i.e., skin-to-skin care or kangaroo care, PC might also include hand-touch only (Kobus et al., 2024). In high-income countries, close PC leads to cardiorespiratory and temperature stability, reduces pain reactions in the preterm infant and promotes sleep organization (Bastani et al., 2017; Durmaz et al., 2023; Pavlyshyn and Sarapuk, 2023). Studies suggest its positive impact on long-term neurodevelopmental outcome (Gonya et al., 2017). In mothers, the beneficial effect of close PC includes facilitated bonding, higher rates of breastfeeding as well as reduced stress levels (Cong et al., 2021; Oras et al., 2016).

Despite the overlapping effects of both MT and PC, recent studies hint at beneficial effects of combining both practices (Teckenberg-Jansson et al., 2011; Yakobson et al., 2021; Kobus et al., 2022b) on preterm infants’ vital parameters, parental well-being and parent-infant-attachment. A recent study by Span et al. found equal beneficial effects of combined live performed MT with kangaroo care on physiological parameters and neurological functioning (Span et al., 2021) in preterm infants compared to MT alone. However, Teckenberg-Jansson et al. showed more beneficial effects of combined MT and kangaroo care compared to kangaroo care alone on infants’ vital parameters and parental well-being (Teckenberg-Jansson et al., 2011).

As part of a randomized-controlled trial (RCT), our group recently showed that MT with parental physical contact (close PC; or hand touch contact) has positive effects on the preterm infants’ vital signs and behavior assessed with the COMFORTneo score, independent of the type of physical contact (Kobus et al., 2024).

A further aim of this RCT and the current study was to evaluate the effect of this intervention on the parental stress level by measuring salivary cortisol as well as self-rated stress levels assessed with the perceived stress questionnaire 20 (PSQ-20).

## 2 Methods

### 2.1 Study design

The aim of this prospective, randomized, controlled clinical trial was to investigate the effects of live performed MT combined with PC on salivary cortisol and perceived stress levels of parents from preterm infants during their NICU stay until 4 weeks after delivery. Study approval was obtained from the local ethics committee of the Medical Faculty of the University of Duisburg-Essen (21-9823-BO). The study was registered with the German registry for clinical studies (registration number: DRKS00025755).

### 2.2 Cohort recruitment

Cohort recruitment of preterm infants (gestational age (GA) < 37 + 0 weeks) started in July 2021 in a level III NICU. The cortisol and stress level analyses were performed as an exploratory add-on study, thus the power analysis relied on the primary outcome of the main study (COMFORTneo score before versus after MT, please see Supplementary material 1) (Kobus et al., 2024). Infants were excluded from eligibility for this study if not stable enough to leave the incubator or cod, or if they participated in a simultaneous interventional trial. Infants were recruited and randomized 1:1 to either standard medical care (SC) or standard medical care plus music therapy with physical contact (MT). The intervention consisted of up to 10 sessions of MT, five each with close PC and five with hand touch only. Written informed consent was obtained from all caregivers prior to inclusion into the study and subsequent randomization. Prior to randomization and the first music therapy intervention, parents had to decide, if mother or father would accompany all therapy sessions. The participating mothers or fathers were asked to weekly collect saliva for cortisol measurements and to answer the PSQ-20 in paper form directly after randomization and for the intervention group before the first session of MT as well as after 4 weeks.

### 2.3 Standard physical contact between parents and preterm infants

Close PC, i.e., skin-to-skin contact or kangaroo care, is an established and standardized procedure on our NICU to promote bonding between parents and preterm infants as well as breast feeding and to enable contact to the parental skin environment. During skin-to-skin contact, the infant is placed naked (only wearing a diaper) on the parental breast/skin. A hand mirror can be used by the parent to better follow the infant’s reactions. In case of (non-)invasive ventilation, the devices are secured properly. The duration of early skin-to-skin contact is set individually. During the NICU stay, skin-to-skin contact should be provided several hours a day and last at least 1 h. The first skin-to-skin contact is desirable within the first 2 h of the infant’s life if mother and infant are stable enough.

### 2.4 Live music therapy intervention

MT was performed as previously described (Kobus et al., 2024). Briefly, MT sessions were performed individually by a trained music therapist using the instrument sansula, which creates vibrating, long-lasting and soft sounds at a low level. The infant’s breathing and reactions guided the sequences. MT sessions were integrated into the clinical routine and timepoints were coordinated between music therapist, parent, and nursing staff. A maximum of 10 MT sessions were performed in clinically stable preterm infants in the presence of the determined parent three to four times per week. MT sessions were performed either with parental hand touch with the infant remaining in the incubator or (heated) cod or with close PC with the infant lying in the parent’s arms, on the chest, legs, or shoulder. The choice between hand touch and close PC sessions was made either according to clinical needs, or the type of contact infant and parent had before the start of MT.

### 2.5 Salivary cortisol analysis

For the assessment of salivary cortisol levels, participants self-collected saliva in the morning after awakening using commercial collection devices (Salivette Cortisol; Sarstedt, Nuembrecht, Germany). To avoid any contamination, they were asked not to eat, drink, smoke or brush teeth before sample collection. Saliva samples were returned to the laboratory, where the saliva was harvested by centrifugation and stored at −80° until analysis. Salivary cortisol concentrations were analyzed by enzyme-linked immunosorbent assay (Cortisol Saliva ELISA, IBL International, Hamburg, Germany) according to the manufacturer’s instructions by trained stuff in a laboratory that regularly takes part in round robin tests for quality assurance and method validation. Cross-reactivity of the anti-cortisol antibody with other relevant steroids was 8.5% (11-deoxycortisol), 2.6% (cortisone), 1.0% (corticosterone), and <0.1% (estrone, estradiol, estriol, progesterone, testosterone). Inter- and intra-assay coefficients of variation were <10%. Salivary cortisol levels were measured weekly over the first 4 weeks. The mothers and fathers collected the cortisol samples irregularly and provided different numbers of samples per week. For the analysis, the mean values of each of the samples per week per parent were determined.

### 2.6 PSQ-20

To quantify the perceived stress level of the participating mother or father before the first session of MT and 4 weeks later, the PSQ-20 was handed out to both the MT and SC group. The short version of the PSQ consists of 20 items, with each item providing a rating on a four-point scale ranging from “almost never,” “sometimes,” “often” to “usually.” The 20 items are assigned to the four subscales “worries,” “tension,” “joy,” and “demands” (Fliege et al., 2009). For each scale, a mean value is calculated. After linear transformation, values range from 0 to 100. For the subscales, high values mean high “worries,” “tension,” “joy,” and “demands.” For the overall score, “joy” items are recoded and a high overall score means high perceived stress [Leibniz Institute for Psychology (ZPID), 2019].

### 2.7 Statistical analyses

Continuous variables are presented as mean with standard deviation (SD) or confidence intervals (CI) if evenly distributed and as median with interquartile range (IQR) if skewed.

If several cortisol probes were submitted per week by the same individual, the average cortisol value per week post-partum was calculated. Mean interindividual cortisol levels were compared by groups and 95% CIs calculated. Effect sizes were calculated according to Hedges (1981) to account for different group sizes.

To estimate the effect of music therapy on cortisol levels, multivariable regression was carried out using a linear mixed model (Supplementary material 2). Fixed effect covariates for adjustment were parents’ sex (categorical) and time since birth (continuous, measured in weeks). To control for multiple measurements within one individual, a “repeated” statement was applied. The repeated statement controls the covariance matrix similar to a random effect but does not produce effect estimates like for a random effect. No interaction terms were used.

PSQ-20 subscores and the total score were calculated according to the original publication (Fliege et al., 2009). Mean subscores and the total scores were calculated with 95% CIs and Cohen’s *d* calculated because the groups had equal sizes.

The interpretation of the effect sizes is based on the following values: large effects with values ≥0.8, medium effects with values of 0.5 and small effects with values of 0.2 (Lakens, 2013).

Analyses were performed and figures produced with SAS Enterprise Guide 8.3 (SAS Institute, Cary, USA).

## 3 Results

### 3.1 Infants and MT sessions

Information on demographic and clinical characteristics of the infants participating in this RCT as well as on MT sessions have previously been published (Kobus et al., 2024). In brief, 100 infants (MT *n* = 50, SC *n* = 50), who met the inclusion criteria for this study were recruited between July 2021 and January 2023. For the analysis of parental parameters, one child of the control group had to be excluded because of early discharge. Mean gestational age was 33.4 weeks (SD 2.0) for the intervention and 31.2 (SD 3.7) for the control group. Mean birth weight was 1904.1 g (SD 479.9) for the intervention and 1604 g (SD 673.7) for the control group. There were 27 male (54%) infants in the intervention and 28 male (57%) infants in the control group. For further details please see Supplementary Table S1. The music therapist performed a total of 486 music therapy sessions with a mean duration of 28.49 min (range 21–33 min). There were 45 infants receiving 10 sessions, four infants receiving eight and one infant receiving four sessions because of early discharge. MT sessions were accompanied by PC, either by close PC or hand touch.

### 3.2 Parental characteristics

**Figure 1:**
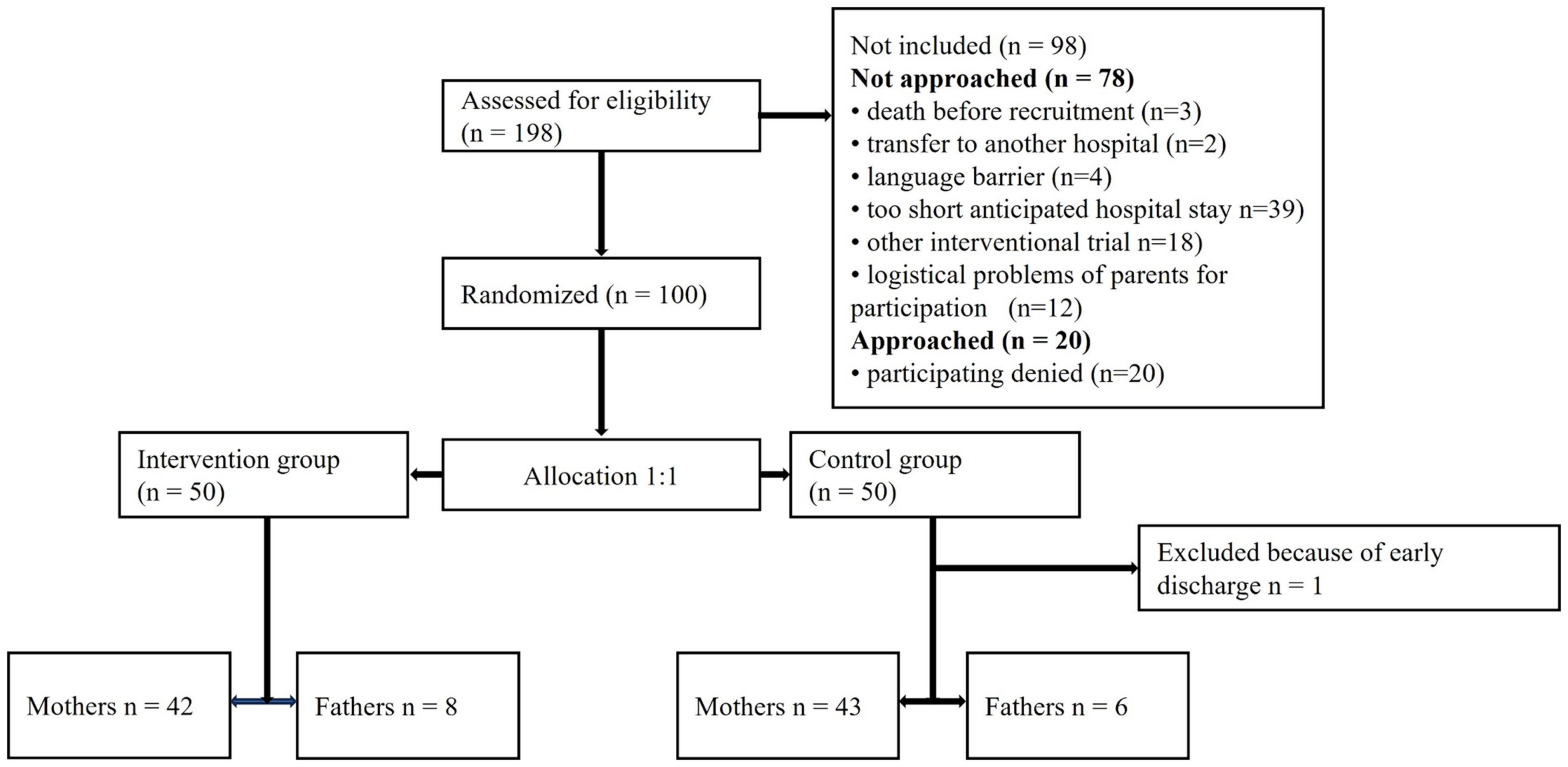
Patient flow.

In the MT group, 42 mothers and 8 fathers committed to accompany all MT sessions, while in the control group 43 mothers and 6 fathers participated in this RCT. Information on demographic and clinical characteristics of all participating parents are presented in Table 1 (mothers) and Table 2 (fathers), and on patient flow in Figure 1. Mothers participated in 406 sessions and fathers in 80 sessions.

**Table 1:** Demographic and clinical characteristics of the included mothers.

	Intervention group (*n* = 42)	Control group (*n* = 43)
Maternal age (mean, SD)	33.6 (5.0)	33.6 (4.8)
Primipara	20 (47.6)	14 (32.6)
Multipara	22 (52.4)	29 (67.4)
Sectio caesarea	33 (78.6)	34 (79.1)
Emergency	3 (7.1)	1 (2.3)
Sec	2 (4.8)	1 (2.3)
Vaginal delivery	4 (9.5)	7 (16.3)
Nicotine abuse	2 (4.8)	0
Drug abuse	1 (2.4)	0
**Pregnancy-related conditions**		
Gestational diabetes	7 (16.7)	4 (9.3)
Preeclampsia	2 (4.8)	3 (7.0)
HELLP	0	0
**Metabolic/endocrine disorders**		
Obesity	7 (16.7)	8 (18.6)
Type 1 diabetes	1 (2.4)	2 (4.6)
Type 2 diabetes	4 (9.5)	4 (9.3)
Hypothyreosis	2 (4.8)	1 (2.3)
**Medication**		
Hydrocortisone	0	1 (2.3)
Prednisolone	1 (2.4)	1 (2.3)

Values are depicted in mean (%), unless otherwise stated. HELLP, hemolysis, elevated liver enzymes, low platelet count.

**Table 2:** Clinical characteristics of the included fathers.

Mean	Intervention group (*n* = 8)	Control group (*n* = 6)
Paternal age (mean, SD)	35.6 (7.0)	34.7 (4.1)
First born child	5 (62.5)	4 (66.7)
Nicotine abuse	0	0
Drug abuse	0	0
**Metabolic/endocrine disorders**		
Obesity	0	1 (16.7)
Type 1 diabetes	1 (12.5)	2 (33.3)
Type 2 diabetes	0	0
Hypothyreosis	0	0
**Medication**		
Hydrocortisone	0	0
Prednisolone	0	0

Values are depicted in mean (%), unless otherwise stated.

### 3.3 Parental salivary cortisol: differences between intervention and control group

**Figure 2:**
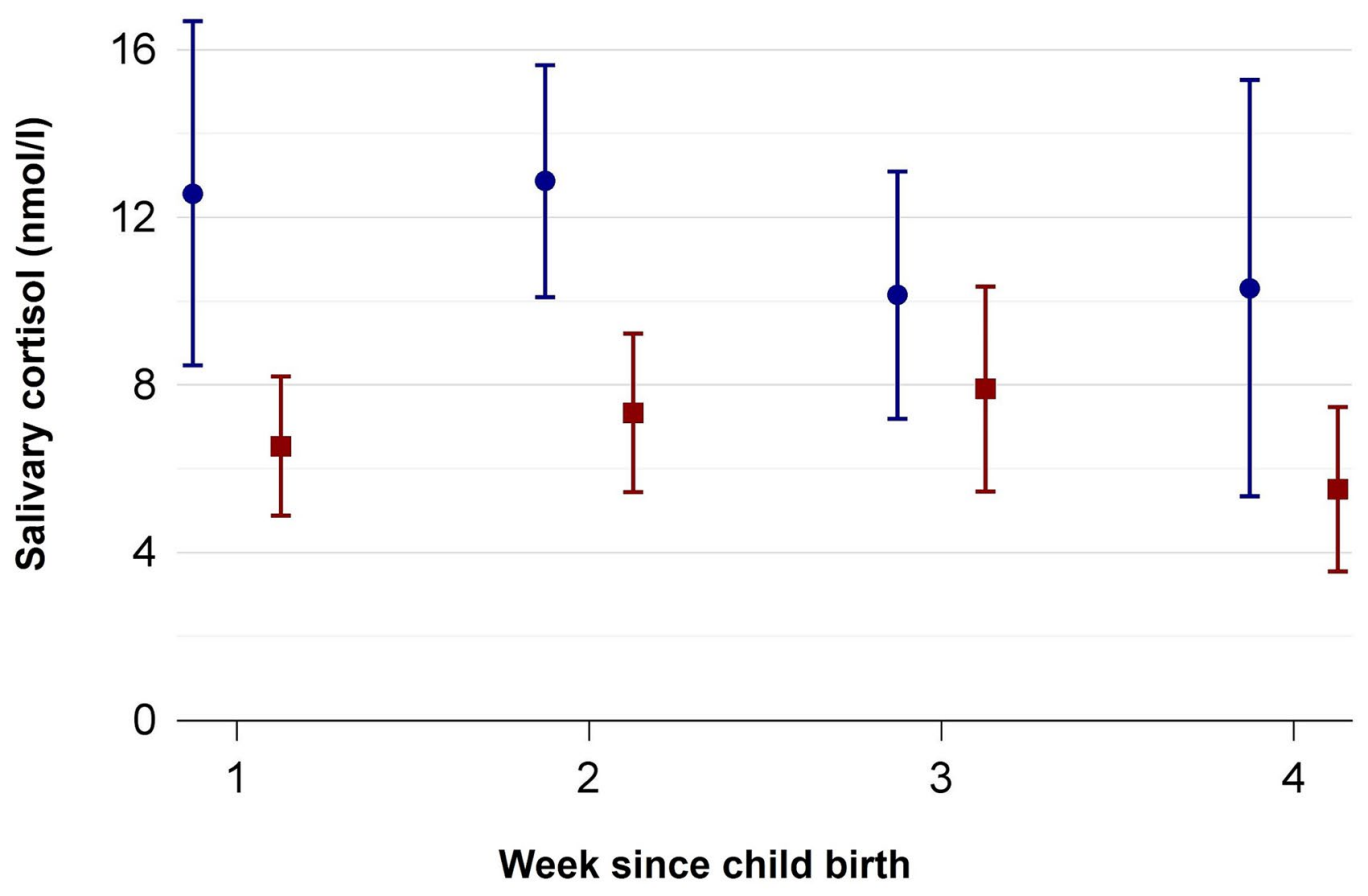
Salivary cortisol measured in nmol/l from week 1 to 4 in mothers and fathers of the standard care (blue circles) and music therapy group (red squares). Mean ± SD.

Two mothers of the control group and one mother of the intervention group had to be excluded from the cortisol analyzes because they received the medication cortisone and prednisolone during the time the samples were collected. While in week 1 *n* = 25 (21 mothers, 4 fathers) of the intervention and *n* = 14 (13 mothers, 1 father) of the control group were analyzed, the return rates declined until week 4 with *n* = 4 samples (3 mothers, 1 fathers) in the intervention group and increased *n* = 11 samples (8 mothers, 3 fathers) in the control group. Supplementary Table S2 shows how many probes (*n*) were analyzed in week 1–4 and the mean, minimum and maximum number of probes per participant. Figure 2 depicts the course of the parental salivary cortisol levels of the intervention and control groups from week 1 to 4, with lower salivary cortisol values on each timepoint for the MT group. Table 3 shows the corresponding mean cortisol levels including the 95% confidence intervals (CI) for the intervention and control group. Hedge’s g effect sizes could only be determined for the mothers, because the groups of participating fathers were too small. In weeks 1, 2 and 4 the Hedge’s effect sizes indicate large effects, while in week 3 an effect size of 0.5 indicates medium effects (Lakens, 2013).

**Table 3:** Parental salivary cortisol levels during the first 4 weeks after recruitment.

Timepoint	Parents	Intervention group		Control group		Effect size^*^
		*N*	Mean (nmol/l) (95% CI)	*N*	Mean (nmol/l) (95% CI)	
Week 1	Mother	21	6.5 (4.9–8.2)	13	12.6 (8.5–16.7)	−1.2
	Father	4	3.7 (0.7–6.6)	1	3.0	^**^
Week 2	Mother	21	7.3 (5.4–9.2)	15	12.9 (10.1–15.6)	−1.2
	Father	5	5.7 (0.9–10.5)	1	9.0	^**^
Week 3	Mother	16	7.9 (5.5–10.4)	13	10.2 (7.2–13.1)	−0.5
	Father	4	8.7 (−0.8–18.2)	2	13.4 (−55.3–82.0)	^**^
Week 4	Mother	7	5.5 (3.6–7.5)	7	10.3 (5.4–15.3)	−1.2
	Father	2	8.3 (7.2–9.4)	3	14.8 (8.9–20.7)	^**^

CI = confidence interval.

* Hedge’s g, control group is considered the reference group.

** too few observations in control/intervention group.

The adjusted mean cortisol levels according to multivariable regression was 6.2 nmol/l (95 CI 4.8–7.7, standard error (SE) 0.7) in the MT group versus 11.6 nmol/l (CI 9.5–13.8, SE 1.1) in the control group. The adjusted difference between the groups was 5.4 nmol/l (3.1–7.7, SE 1.1).

### 3.4 PSQ-20: effect of music therapy with PC on perceived stress in parents over the first 4 weeks after birth

**Figure 3:**
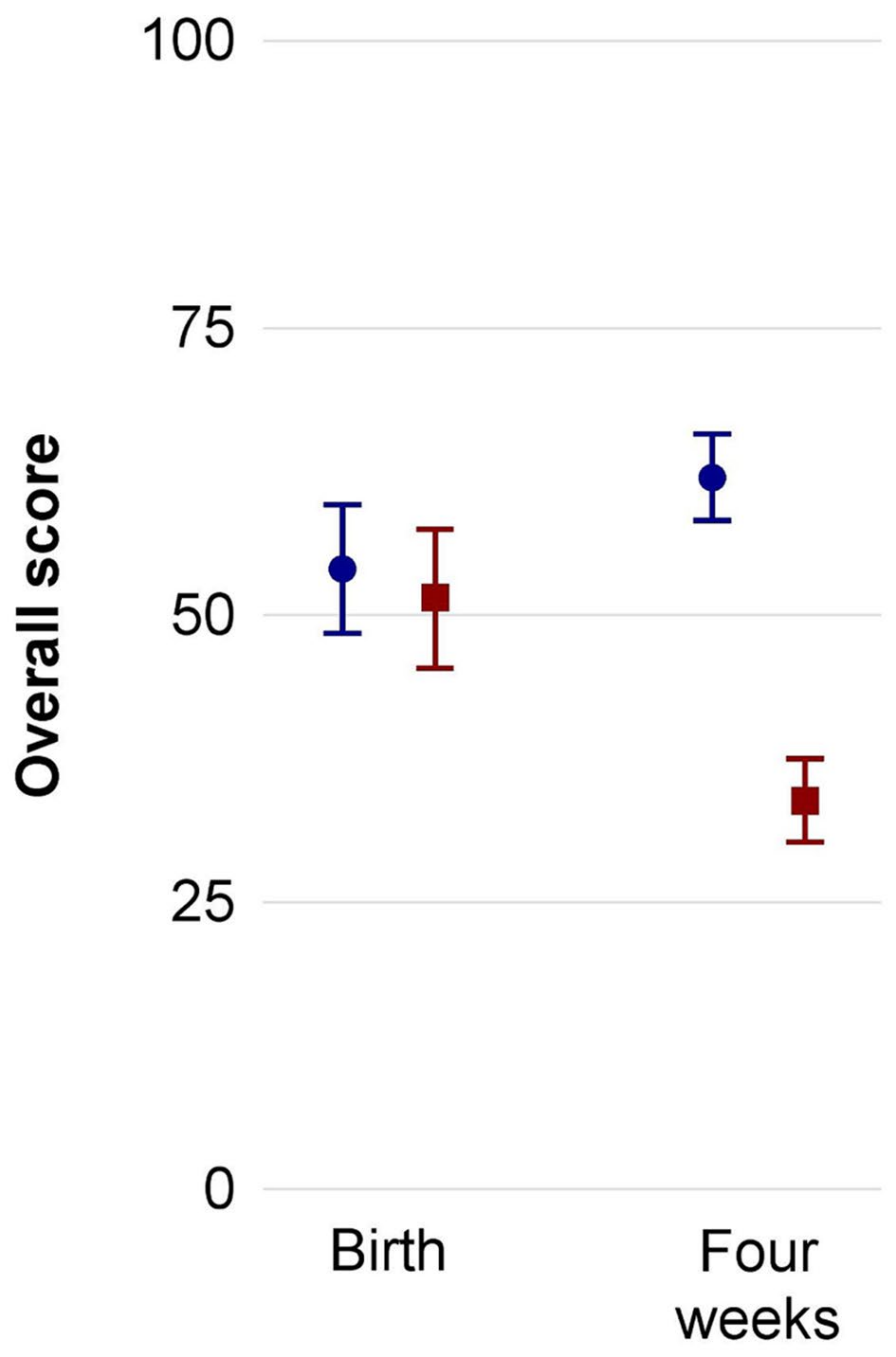
Overall PSQ-20 results including both mothers and fathers of the standard care (blue circles) and music therapy group (red squares) before start of intervention and 4 weeks later. Mean ± SD. PSQ, perceived stress questionnaire.

Figure 3 presents the overall PSQ scores at birth (T1) and 4 weeks later (T2) in mothers and fathers of the intervention group (red squares) and control group (blue circles). Supplementary Figures S1A–D differentiates the four scales of the PSQ-20, that is “worries,” “tension,” “joy,” and “demands.” A subgroup analysis for the mothers is provided in Supplementary Figure S2. The backflow of questionnaires was complete for both groups at both timepoints. The corresponding statistical details on the PSQ-20 results are depicted in Table 4, which includes the mean score and subscores at T1 (before recruitment) and T2 (4 weeks later) as well as the CI for the intervention and control group. Cohen’s *d* effect sizes were determined for T1 and T2. While at the time of birth, overall perceived stress as well as scores for “worries,” “tension,” “joy,” and “demands” were similar between the intervention and control group, reduced overall stress scores were measured 4 weeks later as well as reduced scores in the categories “worries,” “tension,” and “demands” for the intervention group with low effect sizes. For the control group, overall stress was higher after 4 weeks with large effect sizes.

**Table 4:** Total PSQ-20 scores and subscores (95% confidence interval) of parents of preterm infants with and without music therapy before recruitment (T1) and 4 weeks later (T2).

	Parents	Intervention group			Effect size^*^	Control group			Effect size^*^
		*N*	T1 (95% CI)	T2 (95% CI)		*N*	T1 (95% CI)	T2 (95% CI)	
Total Score	Mother	41	51.4 (45.4–57.5)	33.8 (30.0–37.5)	−0.15	43	55.0 (48.8–61.3)	61.1 (57.0–65.2)	−2.19
	Father	9	48.7 (30.0–67.4)	36.1 (22.7–49.5)	0.10	6	46.7 (33.2–60.1)	68.1 (55.7–80.4)	−2.07
Worries	Mother	41	46.4 (39.3–53.5)	23.3 (19.0–27.7)	0.01	43	46.0 (37.0–55.0)	51.5 (45.5–57.5)	−1.72
	Father	9	47.4 (26.2–68.6)	28.1 (12.2–44.1)	0.65	6	32.2 (17.3–47.2)	55.6 (31.5–79.7)	−1.27
Tension	Mother	41	56.1 (48.6–63.7)	35.9 (31.4–40.4)	−0.18	43	61.6 (53.7–69.4)	68.5 (63.6–73.4)	−2.03
	Father	9	52.6 (32.0–73.2)	36.3 (25.4–47.2)	0.08	6	54.4 (37.6–71.2)	77.8 (65.5–90.0)	−3.12
Joy	Mother	41	55.3 (50.2–60.5)	66.7 (62.1–71.2)	0.37	43	48.2 (43.1–53.3)	44.0 (39.4–48.7)	1.69
	Father	9	59.3 (41.8–76.7)	61.5 (41.3–81.6)	0.30	6	53.3 (38.0–68.7)	40.0 (35.6–44.4)	0.77
Demands	Mother	41	58.5 (50.8–66.2)	42.8 (38.0–47.6)	−0.05	43	60.8 (52.9–68.7)	68.5 (64.2–72.8)	−1.67
	Father	9	54.1 (31.7–76.5)	41.5 (27.3–55.7)	0.03	6	53.3 (36.8–69.9)	78.9 (66.1–91.7)	−2.29

CI = confidence interval.

* Cohen’s *d*.

## 4 Discussion

This study on the effect of live-performed MT with PC in preterm infants on parental salivary cortisol and perceived stress levels during the first 4 weeks after birth suggests decreased salivary cortisol and perceived stress levels in the MT group compared to the control group.

With cortisol as objective stress marker, this is, to the authors’ best knowledge, the first study addressing the effect of MT with PC in preterm infants on parental cortisol secretion. The hormone cortisol is used as a biochemical marker of acute and chronic stress. An increase in this hormone as an indicator of stress can be modified by psychosocial interventions like music therapy (Hasanah and Haikal, 2022). Cortisol is released by the hypothalamus-pituitary–adrenal axis in response to stress. The underlying mechanisms of music on cortisol release include the activation of brain circuits involved in pleasure and reward (Blood and Zatorre, 2001). There are connections between hypothalamic nuclei and subcortical structures such as the amygdala (Hasanah and Haikal, 2022; Jensen et al., 2024). Furthermore, music might have a direct inhibiting effect on the Corticotropin-releasing factor in the hypothalamus, which are possible explanations for our results (Bowling, 2023). A salivary cortisol reducing effect of music and singing could already been shown in pregnant women, which was accompanied by improved well-being and mother-infant bonding (Wulff et al., 2021), as well as in mothers of 3–18 months old infants (Fancourt and Perkins, 2018).

Besides the reduced salivary cortisol levels, also subjectively perceived stress was reduced in the MT group compared to the control group after 4 weeks of intervention. In contrast, the perceived stress levels in the control group increased over 4 weeks, possibly due to the increasing participation of the parents in the care of their children during the NICU care, which might cause stress (Buccione et al., 2024).

The stress reducing effect of MT on parents, especially on mothers, has previously been shown by different studies (Kobus et al., 2022a; Palazzi et al., 2021). Only few RCT addressed the effect of MT with PC in preterm infants on parental stress levels. Lai et al. showed that relaxing music (not live-performed) during close PC resulted in lower maternal anxiety compared to close PC alone (Lai et al., 2006). Teckenberg-Jansson et al. also found that dual therapy (live-performed MT and kangaroo care) was more effective in reducing parental stress than kangaroo care alone (Teckenberg-Jansson et al., 2011). A further RCT comparing MT plus skin-to-skin contact to skin-to-skin contact alone showed a non-significant tendency for greater decrease in anxiety levels as well as a trend toward a larger decrease in stress levels in the MT group (Yakobson et al., 2021).

Reducing parental stress levels during the NICU stay is important, as it improves parent-infant bonding and attachment (Ettenberger et al., 2021). A recent study found an increase in breastfeeding rates among mothers who participated in MT sessions compared to control mothers (Vianna et al., 2011). Ak et al. could show in a RCT that recorded music administered to mothers of preterm infants during breast milk expression leads to increased levels of breast milk (Ak et al., 2015). Parental stress, bonding, and breast feeding are important factors which might contribute to improved (neuro-)developmental outcomes in preterm infants (Ettenberger et al., 2021; Sullivan et al., 2023; Lapidaire et al., 2022), although a current RCT on the effect of creative MT in very preterm infants did not find differences in neurodevelopmental outcomes at 24 months corrected age between the intervention and control group (Haslbeck et al., 2023).

This study has some limitations. Due to our study design, the total time of close PC outside MT sessions was not recorded. As close PC is well established on our NICU for years, we interpreted the study results assuming that infants in the MT and control group underwent a similar amount of close PC. Although close PC alone might also reduce parental stress levels (Cong et al., 2021; Pathak et al., 2023), the reinforcing effect of combined MT with PC is suggested by the studies discussed above (Teckenberg-Jansson et al., 2011; Yakobson et al., 2021; Lai et al., 2006). A further limitation of this study is the relatively high dropout rate and differences regarding compliance between the intervention and control group. The bias in the turnover rate in favor of the intervention group may be because parents in the control group were less willing to provide cortisol samples. The drastic decrease in the number of participants providing saliva samples might also be explained by their increasing involvement in the care for their children during the NICU stay (Buccione et al., 2024). Despite the missing values, this study already shows very clear results with large effect sizes (Lakens, 2013). Larger sample sizes are needed to further investigate the effect of MT on parental cortisol levels. Additionally, the results are limited by the fact that cortisol can be influenced by several factors like sleep quality (Hirotsu et al., 2015), menstrual cycle phase (Hamidovic et al., 2020) and body weight (Brix et al., 2021). At least the prevalence of obese parents was similar between the intervention and control group. Cortisol secretion underlies a circadian rhythm, which peaks at around 8 a.m., but participants were instructed to collect saliva directly after waking up in the morning. Despite several factors influencing cortisol levels, salivary cortisol is considered the “gold standard” biomarker for chronic stress and is a valid surrogate of plasma cortisol (Levine et al., 2007; Tammayan et al., 2021). The study results are furthermore limited by the fact that stress level in parents measured with PSQ-20 and salivary cortisol, were only obtained two times and weekly, respectively, because this is an exploratory study on the effect of MT with PC on parental stress. Further time points must be investigated in future studies because stress may fluctuate during the NICU stay due to changing circumstances of the child.

## 5 Conclusion

The results of this study suggest that live-performed MT with PC in preterm infants leads to different parental perceived stress and salivary cortisol levels during the first 4 weeks after birth, potentially mediating beneficial effects for the infant’s cognitive and socio-emotional development. Based on our recent and previous study results, the optimal setting to perform live MT might be in combination with PC. Future studies must investigate the effect of this dual strategy on the long-term development in preterm infants.

## Data Availability

The original contributions presented in the study are included in the article/Supplementary material, further inquiries can be directed to the corresponding author.

## Supplementary material

SUPPLEMENTARY FIGURE S1 PSQ-20 results for the scales “worries,” “tension,” “joy,” and “demands” **(A–D)** of mothers and fathers of the standard care (blue circles) and music therapy group (red squares) before start of intervention and 4 weeks later. Mean ± SD. PSQ, perceived stress questionnaire.

SUPPLEMENTARY FIGURE S2 Overall PSQ-20 results for mothers of the standard care (blue circles) and music therapy group (red squares) before start of intervention and 4 weeks later. Mean ± SD. PSQ, perceived stress questionnaire.

## References

[ref1] Ak J. Lakshmanagowda P. B. G C. M. P. Goturu J. (2015). Impact of music therapy on breast milk secretion in mothers of premature newborns. J. Clin. Diagn. Res. 9:CC04–CC6. doi: 10.7860/JCDR/2015/11642.5776, PMID: 26023551 PMC4437063

[ref2] Bastani F. Rajai N. Farsi Z. Als H. (2017). The effects of kangaroo care on the sleep and wake states of preterm infants. J. Nurs. Res. 25, 231–239. doi: 10.1097/JNR.0000000000000194, PMID: 28481819

[ref3] Bedetti L. Lugli L. Bertoncelli N. Spaggiari E. Garetti E. Lucaccioni L.. (2023). Early skin-to-skin contact in preterm infants: is it safe? An Italian experience. Children 10:570. doi: 10.3390/children10030570, PMID: 36980127 PMC10047376

[ref4] Blood A. J. Zatorre R. J. (2001). Intensely pleasurable responses to music correlate with activity in brain regions implicated in reward and emotion. Proc. Natl. Acad. Sci. USA 98, 11818–11823. doi: 10.1073/pnas.191355898, PMID: 11573015 PMC58814

[ref5] Bowling D. L. (2023). Biological principles for music and mental health. Transl. Psychiatry 13:374. doi: 10.1038/s41398-023-02671-4, PMID: 38049408 PMC10695969

[ref6] Brix J. M. Tura A. Herz C. T. Feder A. Krzizek E. C. Parzer V.. (2021). The association of cortisol excretion with weight and metabolic parameters in nondiabetic patients with morbid obesity. Obes. Facts 14, 510–519. doi: 10.1159/000517766, PMID: 34496367 PMC8546449

[ref7] Buccione E. Scarponcini Fornaro D. Pieragostino D. Natale L. D'Errico A. Chiavaroli V.. (2024). Parents' participation in care during neonatal intensive care unit stay in COVID-19 era: an observational study. Nurs. Rep. 14, 1212–1223. doi: 10.3390/nursrep14020092, PMID: 38804425 PMC11130904

[ref8] Cong S. Wang R. Fan X. Song X. Sha L. Zhu Z.. (2021). Skin- to-skin contact to improve premature mothers' anxiety and stress state: a meta-analysis. Matern. Child Nutr. 17:e13245. doi: 10.1111/mcn.13245, PMID: 34258864 PMC8476413

[ref9] Cong X. Wu J. Vittner D. Xu W. Hussain N. Galvin S.. (2017). The impact of cumulative pain/stress on neurobehavioral development of preterm infants in the NICU. Early Hum. Dev. 108, 9–16. doi: 10.1016/j.earlhumdev.2017.03.003, PMID: 28343092 PMC5444300

[ref10] Cook K. M. De Asis-Cruz J. Kim J. H. Basu S. K. Andescavage N. Murnick J.. (2023). Experience of early-life pain in premature infants is associated with atypical cerebellar development and later neurodevelopmental deficits. BMC Med. 21:435. doi: 10.1186/s12916-023-03141-w, PMID: 37957651 PMC10644599

[ref9001] Dewan M. V. Jungilligens J. Kobus S. Diezel M. Dathe A. K. Schweiger B.. (2024). The effect of live music therapy on white matter microstructure in very preterm infants - A randomized controlled trial. Eur J Paediatr Neurol. 51, 132–139. doi: 10.1016/j.ejpn.2024.06.009 38941879

[ref11] Durmaz A. Sezici E. Akkaya D. D. (2023). The effect of kangaroo mother care or skin-to-skin contact on infant vital signs: a systematic review and meta-analysis. Midwifery 125:103771. doi: 10.1016/j.midw.2023.103771 37454580

[ref12] Erdei C. Sunwoo J. Corriveau G. C. Forde M. El-Dib M. Inder T. (2024). Effect of music- based interventions on physiologic stability of hospitalized preterm infants. A pilot study. J. Perinatol. 44, 665–670. doi: 10.1038/s41372-024-01907-5, PMID: 38418527

[ref13] Ettenberger M. Bieleninik Ł. Epstein S. Elefant C. (2021). Defining attachment and bonding: overlaps, differences and implications for music therapy clinical practice and research in the neonatal intensive care unit (NICU). Int. J. Environ. Res. Public Health 18:1733. doi: 10.3390/ijerph18041733, PMID: 33579015 PMC7916808

[ref14] Fancourt D. Perkins R. (2018). The effects of mother–infant singing on emotional closeness, affect, anxiety, and stress hormones. Music Sci. 1:205920431774574. doi: 10.1177/2059204317745746

[ref15] Fernández Medina I. M. Granero-Molina J. Fernández-Sola C. Hernández-Padilla J. M. Camacho Ávila M. López Rodríguez M. D. M. (2018). Bonding in neonatal intensive care units: experiences of extremely preterm infants' mothers. Women Birth 31, 325–330. doi: 10.1016/j.wombi.2017.11.008, PMID: 29191725

[ref16] Fliege H. Rose M. Arck P. Levenstein S. Klapp B. F. (2009). “PSQ. Perceived Stress Questionnaire [Verfahrensdokumentation aus PSYNDEX Tests-Nr. 9004426, PSQ20- Skalenberechnung, PSQ20-Fragebogen Englisch, Deutsch, Deutsch (letzte 2 Jahre), PSQ30- Skalenberechnung, PSQ30-Fragebogen Englisch, Französisch, Deutsch, Italienisch, und Spanisch]. In Leibniz-Zentrum für Psychologische Information und Dokumentation (ZPID) (Hrsg.)” in Elektronisches Testarchiv (Trier: ZPID).

[ref17] Gonya J. Ray W. C. Rumpf R. W. Brock G. (2017). Investigating skin-to-skin care patterns with extremely preterm infants in the NICU and their effect on early cognitive and communication performance: a retrospective cohort study. BMJ Open 7:e012985. doi: 10.1136/bmjopen-2016-012985, PMID: 28320787 PMC5372108

[ref18] Hamidovic A. Karapetyan K. Serdarevic F. Choi S. H. Eisenlohr-Moul T. Pinna G. (2020). Higher circulating cortisol in the follicular vs. luteal phase of the menstrual cycle: a Meta-analysis. Front. Endocrinol. 11:311. doi: 10.3389/fendo.2020.00311, PMID: 32582024 PMC7280552

[ref19] Hasanah I. Haikal Z. (2022). The effects of music therapy on cortisol levels as a biomarker of stress in children [Internet]. Music in Health and Diseases. IntechOpen. doi: 10.5772/intechopen.99734

[ref20] Haslbeck F. B. Adams M. Schmidli L. Bassler D. Bucher H. U. Natalucci G. (2023). Creative music therapy for long-term neurodevelopment in extremely preterm infants: results of a feasibility trial. Acta Paediatr. 112, 2524–2531. doi: 10.1111/apa.16984, PMID: 37787033

[ref21] Haslbeck F. B. Bucher H. U. Bassler D. Hagmann C. Natalucci G. (2021). Creative music therapy and neurodevelopmental outcomes in pre-term infants at 2 years: a randomized controlled pilot trial. Front. Pediatr. 9:660393. doi: 10.3389/fped.2021.660393, PMID: 34222141 PMC8249730

[ref22] Haslbeck F. B. Jakab A. Held U. Bassler D. Bucher H. U. Hagmann C. (2020). Creative music therapy to promote brain function and brain structure in preterm infants: a randomized controlled pilot study. NeuroImage Clin. 25:102171. doi: 10.1016/j.nicl.2020.102171, PMID: 31972397 PMC6974781

[ref23] Hedges L. V. (1981). Distribution theory for Glass’s estimator of effect size and related estimators. J. Educ. Stat. 6, 107–128. doi: 10.3102/10769986006002107

[ref24] Hirotsu C. Tufik S. Andersen M. L. (2015). Interactions between sleep, stress, and metabolism: from physiological to pathological conditions. Sleep Sci 8, 143–152. doi: 10.1016/j.slsci.2015.09.002, PMID: 26779321 PMC4688585

[ref25] Jensen D. E. A. Ebmeier K. P. Suri S. Rushworth M. F. S. Klein-Flügge M. C. (2024). Nuclei-specific hypothalamus networks predict a dimensional marker of stress in humans. Nat. Commun. 15:2426. doi: 10.1038/s41467-024-46275-y, PMID: 38499548 PMC10948785

[ref26] Kehl S. M. La Marca-Ghaemmaghami P. Haller M. Pichler-Stachl E. Bucher H. U. Bassler D.. (2020). Creative music therapy with premature infants and their parents: a mixed-method pilot study on parents' anxiety, stress and depressive symptoms and parent- infant attachment. Int. J. Environ. Res. Public Health 18:265. doi: 10.3390/ijerph18010265, PMID: 33396496 PMC7795112

[ref27] Khoramirad A. Abedini Z. Khalajinia Z. (2021). Relationship between mindfulness and maternal stress and mother – infant bonding in neonatal intensive care unit. J. Educ. Health Promot. 10:337. doi: 10.4103/jehp.jehp_1620_20, PMID: 34761023 PMC8552262

[ref28] Kobus S. Diezel M. Dewan M. V. Huening B. Dathe A. K. Felderhoff-Mueser U.. (2021). Music therapy is effective during sleep in preterm infants. Int. J. Environ. Res. Public Health 18:8245. doi: 10.3390/ijerph18168245, PMID: 34443994 PMC8391215

[ref29] Kobus S. Diezel M. Dewan M. V. Huening B. Dathe A. K. Felderhoff-Mueser U.. (2022b). Impact of physical contact on preterm Infants' vital sign response to live music therapy. Int. J. Environ. Res. Public Health 19:9524. doi: 10.3390/ijerph19159524, PMID: 35954880 PMC9368366

[ref30] Kobus S. Diezel M. Dewan M. V. Huening B. Dathe A. K. Marschik P. B.. (2022a). Music therapy in preterm infants reduces maternal distress. Int. J. Environ. Res. Public Health 20:731. doi: 10.3390/ijerph20010731, PMID: 36613052 PMC9819311

[ref31] Kobus S. Diezel M. Dewan M. V. Huening B. Dathe A. K. Marschik P. B.. (2023). Music therapy modulates mothers' perception of their preterm infants. Front. Psychol. 14:1231741. doi: 10.3389/fpsyg.2023.1231741, PMID: 37928582 PMC10620800

[ref32] Kobus S. Kleinbeck T. Ader M. Dewan M. V. Dathe A. K. Feddahi N.. (2024). COMFORTneo scale in preterm infants during live performed music therapy-difference between close physical contact and hand touch contact. Front. Neurosci. 18:1359769. doi: 10.3389/fnins.2024.1359769, PMID: 38606306 PMC11008230

[ref33] Kraft K. E. Jaschke A. C. Ravensbergen A. G. Feenstra-Weelink A. van Goor M. E. L. de Kroon M. L. A.. (2021). Maternal anxiety, infant stress, and the role of live-performed music therapy during NICU stay in the Netherlands. Int. J. Environ. Res. Public Health 18:7077. doi: 10.3390/ijerph18137077, PMID: 34281014 PMC8297304

[ref34] Lai H. L. Chen C. J. Peng T. C. Chang F. M. Hsieh M. L. Huang H. Y.. (2006). Randomized controlled trial of music during kangaroo care on maternal state anxiety and preterm infants' responses. Int. J. Nurs. Stud. 43, 139–146. doi: 10.1016/j.ijnurstu.2005.04.008, PMID: 15996669

[ref35] Lakens D. (2013). Calculating and reporting effect sizes to facilitate cumulative science: a practical primer for t-tests and ANOVAs. Front. Psychol. 4:863. doi: 10.3389/fpsyg.2013.00863, PMID: 24324449 PMC3840331

[ref36] Lapidaire W. Lucas A. Clayden J. D. Clark C. Fewtrell M. S. (2022). Human milk feeding and cognitive outcome in preterm infants: the role of infection and NEC reduction. Pediatr. Res. 91, 1207–1214. doi: 10.1038/s41390-021-01367-z, PMID: 34168271 PMC9122812

[ref37] Leibniz Institute for Psychology (ZPID). (2019). Open test archive: PSQ. Perceived stress questionnaire

[ref38] Levine A. Zagoory-Sharon O. Feldman R. Lewis J. G. Weller A. (2007). Measuring cortisol in human psychobiological studies. Physiol. Behav. 90, 43–53. doi: 10.1016/j.physbeh.2006.08.025 17055006

[ref39] Lundqvist P. Weis J. Sivberg B. (2019). Parents' journey caring for a preterm infant until discharge from hospital-based neonatal home care-a challenging process to cope with. J. Clin. Nurs. 28, 2966–2978. doi: 10.1111/jocn.14891, PMID: 31017322

[ref40] Marlow N. Bennett C. Draper E. S. Hennessy E. M. Morgan A. S. Costeloe K. L. (2014). Perinatal outcomes for extremely preterm babies in relation to place of birth in England: the EPICure 2 study. Arch. Dis. Child. Fetal Neonatal Ed. 99, F181–F188. doi: 10.1136/archdischild-2013-305555, PMID: 24604108 PMC3995269

[ref41] Nusinovici S. Olliac B. Flamant C. Müller J. B. Olivier M. Rouger V.. (2017). Impact of preterm birth on parental separation: a French population-based longitudinal study. BMJ Open 7:e017845. doi: 10.1136/bmjopen-2017-017845, PMID: 29150469 PMC5701975

[ref42] Ohuma E. O. Moller A. B. Bradley E. Chakwera S. Hussain-Alkhateeb L. Lewin A.. (2023). National, regional, and global estimates of preterm birth in 2020, with trends from 2010: a systematic analysis. Lancet 402, 1261–1271. doi: 10.1016/S0140-6736(23)00878-4, PMID: 37805217

[ref43] Oras P. Thernström Blomqvist Y. Hedberg Nyqvist K. Gradin M. Rubertsson C. Hellström-Westas L.. (2016). Skin-to-skin contact is associated with earlier breastfeeding attainment in preterm infants. Acta Paediat. 105, 783–789. doi: 10.1111/apa.13431, PMID: 27100380

[ref44] Palazzi A. Meschini R. Piccinini C. A. (2021). NICU music therapy effects on maternal mental health and preterm infant's emotional arousal. Infant Ment. Health J. 42, 672–689. doi: 10.1002/imhj.21938, PMID: 34378804

[ref45] Pathak B. G. Sinha B. Sharma N. Mazumder S. Bhandari N. (2023). Effects of kangaroo mother care on maternal and paternal health: systematic review and meta-analysis. Bull. World Health Organ. 101, 391–402. doi: 10.2471/BLT.22.288977, PMID: 37265678 PMC10225947

[ref46] Pavlyshyn H. Sarapuk I. (2023). Skin-to-skin contact-an effective intervention on pain and stress reduction in preterm infants. Front. Pediatr. 11:1148946. doi: 10.3389/fped.2023.1148946, PMID: 37033163 PMC10073438

[ref47] Rusanen E. Lahikainen A. R. Vierikko E. Pölkki P. Paavonen E. J. (2024). A longitudinal study of maternal postnatal bonding and psychosocial factors that contribute to social-emotional development. Child Psychiatry Hum. Dev. 55, 274–286. doi: 10.1007/s10578-022-01398-5, PMID: 35870058 PMC10796530

[ref48] Sa de Almeida J. Lordier L. Zollinger B. Kunz N. Bastiani M. Gui L.. (2020). Music enhances structural maturation of emotional processing neural pathways in very preterm infants. NeuroImage 207:116391. doi: 10.1016/j.neuroimage.2019.116391, PMID: 31765804

[ref49] Span L. C. van Dokkum N. H. Ravensbergen A. G. Bos A. F. Jaschke A. C. (2021). Combining kangaroo care and live-performed music therapy: effects on physiological stability and neurological functioning in extremely and very preterm infants. Int. J. Environ. Res. Public Health 18:6580. doi: 10.3390/ijerph18126580, PMID: 34207310 PMC8296373

[ref50] Sullivan G. Vaher K. Blesa M. Galdi P. Stoye D. Q. Quigley A. J.. (2023). Breast milk exposure is associated with cortical maturation in preterm infants. Ann. Neurol. 93, 591–603. doi: 10.1002/ana.26559, PMID: 36412221

[ref51] Tammayan M. Jantaratnotai N. Pachimsawat P. (2021). Differential responses of salivary cortisol, amylase, and chromogranin a to academic stress. PLoS One 16:e0256172. doi: 10.1371/journal.pone.0256172, PMID: 34383867 PMC8360508

[ref52] Teckenberg-Jansson P. Huotilainen M. Polkki T. Lipsanen J. Järvenpää A.-L. (2011). Rapid effects of neonatal music therapy combined with kangaroo care on prematurely-born infants. Nord. J. Music. Ther. 20, 22–42. doi: 10.1080/08098131003768123

[ref53] Vianna M. N. Barbosa A. P. Carvalhaes A. S. Cunha A. J. (2011). Music therapy may increase breastfeeding rates among mothers of premature newborns: a randomized controlled trial. J. Pediatr. 212. doi: 10.2223/JPED.2086, PMID: 21461451

[ref54] Wallois F. Routier L. Bourel-Ponchel E. (2020). Impact of prematurity on neurodevelopment. Handb. Clin. Neurol. 173, 341–375. doi: 10.1016/B978-0-444-64150-2.00026-5 32958184

[ref55] Weigl T. Schneider N. Stein A. Felderhoff-Müser U. Schedlowski M. Engler H. (2020). Postpartal affective and endocrine differences between parents of preterm and full-term infants. Front. Psychiatry. 11:251. doi: 10.3389/fpsyt.2020.00251, PMID: 32296356 PMC7139630

[ref56] Winston R. Chicot R. (2016). The importance of early bonding on the long-term mental health and resilience of children. London J. Prim. Care 8, 12–14. doi: 10.1080/17571472.2015.1133012, PMID: 28250823 PMC5330336

[ref57] Wulff V. Hepp P. Wolf O. T. Balan P. Hagenbeck C. Fehm T.. (2021). The effects of a music and singing intervention during pregnancy on maternal well-being and mother- infant bonding: a randomised, controlled study. Arch. Gynecol. Obstet. 303, 69–83. doi: 10.1007/s00404-020-05727-8, PMID: 32776296 PMC7854426

[ref58] Yakobson D. Gold C. Beck B. D. Elefant C. Bauer-Rusek S. Arnon S. (2021). Effects of live music therapy on autonomic stability in preterm infants: a cluster-randomized controlled trial. Children 8:1077. doi: 10.3390/children8111077, PMID: 34828790 PMC8618386

[ref59] Yue W. Han X. Luo J. Zeng Z. Yang M. (2021). Effect of music therapy on preterm infants in neonatal intensive care unit: systematic review and meta-analysis of randomized controlled trials. J. Adv. Nurs. 77, 635–652. doi: 10.1111/jan.14630, PMID: 33200833

[ref60] Zhang X. Spear E. Hsu H. L. Gennings C. Stroustrup A. (2022). NICU-based stress response and preterm infant neurobehavior: exploring the critical windows for exposure. Pediatr. Res. 92, 1470–1478. doi: 10.1038/s41390-022-01983-3, PMID: 35173301 PMC9378765

