# Supplementary figures and images for: The effect of live-performed music therapy with physical contact in preterm infants on parental perceived stress and salivary cortisol levels
Source: Front Psychol. 2024 Oct 7;15:1441824. doi: 10.3389/fpsyg.2024.1441824 (PMC11492995; doi:10.3389/fpsyg.2024.1441824)

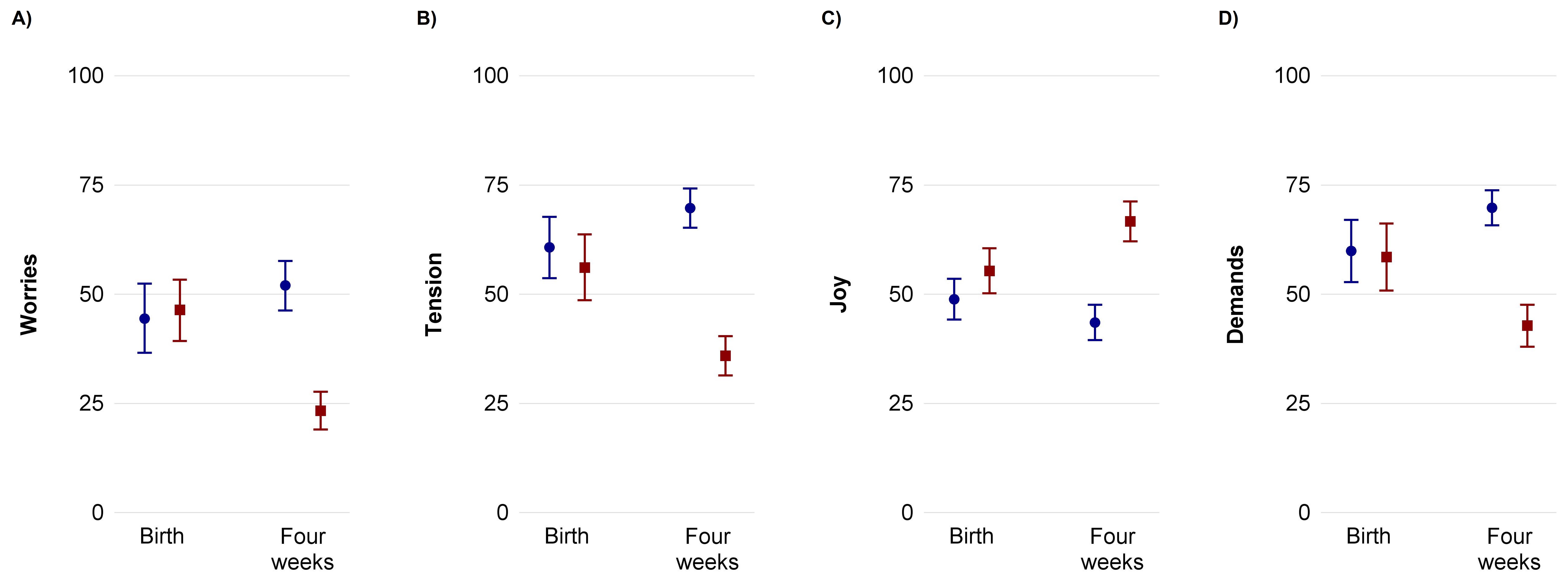

Supplement: SUPPLEMENTARY FIGURE S1 — PSQ-20 results for the scales “worries,” “tension,” “joy,” and “demands” (A–D) of mothers and fathers of the standard care (blue circles) and music therapy group (red squares) before start of intervention and 4 weeks later. Mean ± SD. PSQ, perceived stress questionnaire. [file Image_1.JPEG]

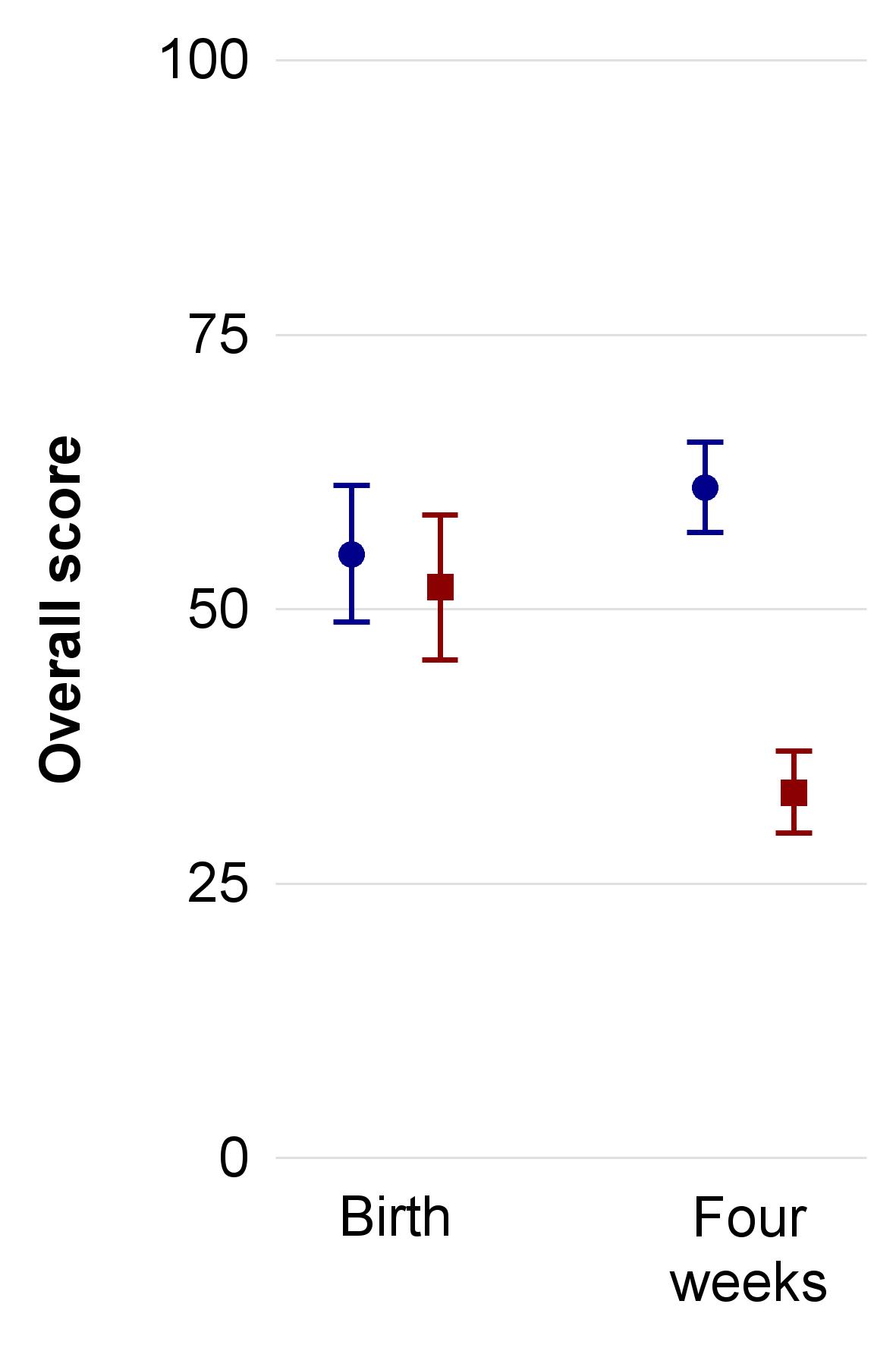

Supplement: SUPPLEMENTARY FIGURE S2 — Overall PSQ-20 results for mothers of the standard care (blue circles) and music therapy group (red squares) before start of intervention and 4 weeks later. Mean ± SD. PSQ, perceived stress questionnaire. [file Image_2.JPEG]
