# Supplementary material for: The effect of live-performed music therapy with physical contact in preterm infants on parental perceived stress and salivary cortisol levels
Source: Front Psychol. 2024 Oct 7;15:1441824. doi: 10.3389/fpsyg.2024.1441824 (PMC11492995; doi:10.3389/fpsyg.2024.1441824)
Supplement: Supplementary file 3 [file Table_1.DOCX]

**Table S1.** Clinical characteristics of the parents’ preterm infants.

|  | **Intervention group**  **(n = 50)** | **Control group**  **(n = 49)** |
| --- | --- | --- |
| Male, n (%) | 27 (54) | 28 (57) |
| GA, weeks | 33.4 (±2.0) | 31.2 (±3,7) |
| GA, weeks, range | 26+0–36+0 | 23+3–36+7 |
| Birth weight, g | 1904 (±480) | 1604 (±674) |
| Birth weight, g, range | 425–2985 | 455–3630 |
| Length at birth, g | 43.0 (±4.3) | 40.5 (±5.9) |
| Length at birth, g, range | 26.0–50.0 | 26.0–51.0 |
| Head circumference at birth, g | 30.8 (±2.4) | 28.8 (±3.7) |
| Head circumference at birth, g, range | 21.5–34.0 | 19.5–35.0 |
| APGAR score at 1 min. | 6.8 (±0.6) | 6.7 (±2.2) |
| APGAR score at 1 min, range | 0.0–10.0 | 1.0–10.0 |
| APGAR score at 5 min. | 8.1 (±0.5) | 7.7 (±1.7) |
| APGAR score at 5 min, range | 1.0–10.0 | 1.0–10.0 |
| APGAR score at 10 min. | 8.9 (±0.4) | 8.5 (±1.5) |
| APGAR score at 10 min, range | 4.0–10.0 | 1.0–10.0 |
| Weight at discharge, g | 2684 (±677) | 2865 (±697) |
| Weight at discharge, g, range | 1955–4880 | 2100–5870 |
| Length at discharge, g | 47.9 (±3.3) | 48.9 (±3.5) |
| Length at discharge, g, range | 43.0–60.0 | 47.9–48.8 |
| Head circumference at discharge, g | 33.4 (±1.9) | 33.8 (±1.6) |
| Head circumference at discharge, g, range | 31.0–39.0 | 31.0–37.6 |
| Antibiotic treatment, days | 3.0 (±5.1) | 2.6 (±5.3) |
| Antibiotic treatment, days, range | 0-26 | 0–31 |

*Notes.* GA = gestational age. Data are presented as mean and standard deviation, if not indicated otherwise.
