# Supplementary material for: The effect of live-performed music therapy with physical contact in preterm infants on parental perceived stress and salivary cortisol levels
Source: Front Psychol. 2024 Oct 7;15:1441824. doi: 10.3389/fpsyg.2024.1441824 (PMC11492995; doi:10.3389/fpsyg.2024.1441824)
Supplement: Supplementary file 4 [file Table_2.DOCX]

**Table S2. Details on cortisol probes backflow.**

| **Parents** | **Group** | **Week** | **N** | **Mean** | **Minimum** | **Maximum** |
| --- | --- | --- | --- | --- | --- | --- |
| Father | Control Group | 1 | 1 | 1.0 | 1.0 | 1.0 |
|  |  | 2 | 1 | 5.0 | 5.0 | 5.0 |
|  |  | 3 | 2 | 2.5 | 1.0 | 4.0 |
|  |  | 4 | 3 | 5.7 | 5.0 | 7.0 |
|  | Intervention Group | 1 | 4 | 2.8 | 1.0 | 5.0 |
|  |  | 2 | 6 | 4.8 | 1.0 | 7.0 |
|  |  | 3 | 5 | 3.4 | 1.0 | 7.0 |
|  |  | 4 | 3 | 1.0 | 1.0 | 1.0 |
| Mother | Control Group | 1 | 13 | 1.8 | 1.0 | 4.0 |
|  |  | 2 | 16 | 4.0 | 1.0 | 7.0 |
|  |  | 3 | 14 | 4.0 | 1.0 | 7.0 |
|  |  | 4 | 9 | 4.7 | 2.0 | 7.0 |
|  | Intervention Group | 1 | 21 | 2.3 | 1.0 | 6.0 |
|  |  | 2 | 22 | 4.6 | 1.0 | 7.0 |
|  |  | 3 | 17 | 3.7 | 1.0 | 7.0 |
|  |  | 4 | 7 | 3.7 | 1.0 | 7.0 |

Analyzed probes (*n*) in week 1-4 and the mean, minimum and maximum number of probes per participant of the control and intervention group.
