# Supplementary material for: The effect of live-performed music therapy with physical contact in preterm infants on parental perceived stress and salivary cortisol levels
Source: Front Psychol. 2024 Oct 7;15:1441824. doi: 10.3389/fpsyg.2024.1441824 (PMC11492995; doi:10.3389/fpsyg.2024.1441824)
Supplement: Supplementary file 5 [file Data_Sheet_1.docx]

**Supplement 1.** Important parameters of power analysis protocol

Effect size 0.3

alpha level 0.05

power 0.8

planned statistic analysis: difference between two dependent means

two tailed
