# Supplementary material for: The effect of live-performed music therapy with physical contact in preterm infants on parental perceived stress and salivary cortisol levels
Source: Front Psychol. 2024 Oct 7;15:1441824. doi: 10.3389/fpsyg.2024.1441824 (PMC11492995; doi:10.3389/fpsyg.2024.1441824)
Supplement: Supplementary file 6 [file Data_Sheet_2.docx]

**Supplement 2.** SAS code

proc mixed data=dataset;
    class mother music_therapy ID;
    model cortisol_level = music_therapy mother time_cat;
    repeated / subject=ID type=sp(pow)(time_cat) group=music_therapy;
    lsmeans music_therapy / at means cl pdiff=all;
run;

**Explanation of variables:**

Mother = binary variable on parent (mother = 1, father = 0)

Music_therapy = binary variable on intervention group (music therapy = 1, standard care = 0)

ID = individual study ID for each patient, necessary to correct for repeated measures

Time_cat = continuous variable on time since birth (in weeks)
